# Variation in Everyday Maternal Sensitivity Predicts Infants’ Real‐Time Physiological Regulation

**DOI:** 10.1002/dev.70193

**Published:** 2026-08-27

**Authors:** Anna Madden‐Rusnak, Megan Micheletti, Taylor Wilds, Kaya de Barbaro

**Affiliations:** ^1^ Department of Women's Health Dell Medical School at the University of Texas at Austin Austin Texas USA; ^2^ Semel Institute for Neuroscience and Human Behavior University of California Los Angeles Los Angeles California USA; ^3^ Department of Psychology the University of Texas at Austin Austin Texas USA

**Keywords:** infant regulation, maternal sensitivity, naturalistic paradigms, RSA, wearable studies

## Abstract

While maternal sensitivity is generally considered a stable trait, recent work has found that caregiver sensitivity varies substantially across the day as caregivers respond to their infants amid everyday demands and distractions. It remains unknown how variation in everyday caregiver sensitivity influences infants’ real‐time physiological regulation during naturally occurring distress. In a sample of 23 infants (1–10 months old, 48% female), we synchronized infant‐worn full‐day home audio recordings and heart rate monitors to examine changes in infants’ respiratory sinus arrhythmia (RSA) following the onset of spontaneous distress, that is, episodes of fussing or crying, occurring across the day (*N* = 332 episodes, *M* = 14.34 per dyad). Caregiver sensitivity was coded individually for each episode of infant distress on a 1–7 scale, and RSA was time‐locked to distress onset. Between‐person analyses found that infants of caregivers with above‐average sensitivity (i.e., sensitivity greater than the sample mean) showed adaptive decreases in RSA following distress onset. Additionally, within‐person analyses found that infants also displayed significant decreases in RSA only on occasions when caregivers responded more sensitively relative to their *own* average. These findings demonstrate that both stable differences in caregiving quality and moment‐to‐moment variation in sensitivity shape infant's physiological regulation during everyday distress interactions.

## 1 Introduction

Caregiver sensitivity, defined as the accurate perception of and timely, appropriate response to a child's cues, particularly to signals of distress such as fussing and crying, plays a foundational role in shaping early emotion regulation and long‐term developmental outcomes (Ainsworth 1969; Newman et al. 2015; World Health Organization 2004; Tsivos et al. 2015). One way researchers have examined the interplay between caregiving and infant regulation is by characterizing infants’ physiological responses across emotional contexts that differ in the degree of caregiver support they require. Physiological measures therefore provide an objective metric of how caregiving shapes emerging regulatory skills. A growing body of research has shown that sensitive caregiving is associated with more adaptive physiological responses to stress in infancy, including distinct patterns of parasympathetic regulation, indexed by respiratory sinus arrhythmia (RSA) (Ahnert et al. 2021; Bosquet Enlow et al. 2014; Conradt and Ablow 2010; Guttmann‐Steinmetz and Crowell 2006; Propper et al. 2008). In particular, decreases in RSA during stressful or challenging contexts—reflecting parasympathetic, or vagal, suppression—are thought to represent adaptive mobilization of physiological resources that support effective regulation of arousal (Bazhenova et al. 2001; Jones‐Mason et al. 2018; Porges 1995; Qu and Leerkes 2018; Sacrey et al. 2021). Consistent with this framework, studies using structured laboratory paradigms, such as the face‐to‐face‐still‐face paradigm, have demonstrated that infants with more sensitive caregivers exhibit more adaptive physiological responses to stress (Conradt and Ablow 2010; Moore et al. 2009). Longitudinal work further suggests that sensitive caregiving can promote resilience, supporting more favorable developmental outcomes among infants who may be at heightened risk for later regulatory difficulties (Brown et al. 2025). Together, this literature highlights the importance of caregiving sensitivity as a key predictor of the expression and developmental trajectory of infant physiological regulation.

At the same time, most of this work has been conducted in structured laboratory settings that capture only a narrow slice of infants’ daily experiences. In these paradigms, caregiver–infant interactions are typically brief, highly controlled, and often constrained in ways that restrict natural responses. At home, infants cry across a diverse range of routines and caregiving contexts: sometimes as a caregiver is changing their diaper, while other times may be as caregivers are attending to other siblings, engaging in work or household chores, or distracted by ongoing media (Branger et al. 2019; Calkins et al. 2001; de Barbaro and Madden‐Rusnak 2026). The everyday contexts of infant distress have been shown to predict variability in caregiver sensitivity across the day, with potential implications for infant regulation (de Barbaro and Madden‐Rusnak 2026). Without capturing the real‐time effects of heterogenous, unstructured caregiving interactions on infant regulation, we cannot fully understand the processes that give rise to adaptive—or maladaptive—patterns of regulation in the environments where they spend most of their time. Accordingly, the present study uses infant‐worn audio recordings synchronized with heart rate monitors to characterize infants’ real‐time vagal regulation following the onset of spontaneous distress, namely, episodes of fussing and crying, amid varying caregiving responses across the day.

### 1.1 Lessons and Limitations of Traditional Assessments

A substantial body of work based on structured laboratory paradigms has yielded important insights into the associations between sensitive caregiving and physiological regulation and the influences of the context in which these interactions are observed. For example, caregiver sensitivity assessed during infant distress paradigms (e.g., still‐face, strange situation) predicts infant RSA baseline and recovery patterns, whereas sensitivity measured in non‐distress contexts does not show the same associations (Leerkes et al. 2009; Tacana et al. 2024). Similarly, research with young children indicates that parasympathetic regulation is most predictive of behavioral outcomes (i.e., better inhibitory control) when measured within caregiver–child interactions rather than in isolation or baseline conditions (Skowron et al. 2014). Together, these findings suggest that caregiving exerts its strongest influence on regulatory processes during moments of distress, when infants are most physiologically reactive.

Despite these critical insights on the importance of context, our empirical understanding of caregiver sensitivity is primarily based on brief and/or structured observations in controlled settings. Sensitivity assessments typically occur during short (5–15 min) video‐recorded free play interactions where infant distress is rare (Leerkes et al. 2009; McElwain and Booth‐LaForce 2006), or in proximity to experimentally induced distress tasks such as the arm restraint or the still‐face paradigm (Bohr et al. 2018; Tryphonopoulos et al. 2016). In parallel, infant physiology is most often indexed using brief (1–5 min) baseline, reactivity, and recovery phases. In these paradigms, the goal is to observe decreases in RSA (vagal suppression) during stress and return to baseline RSA levels during reunion (indicating vagal withdrawal or parasympathetic recovery).

These standardized paradigms impose constraints that may limit ecological validity. For example, in many distress‐inducing assessments, caregivers and infants are positioned face‐to‐face at a fixed distance, allowing for immediate and uninterrupted responses. In contrast, in everyday environments, caregivers may experience delays or barriers in responding due to physical separation or competing demands, resulting in longer and more variable response latencies (Micheletti et al. 2025). Further, structured tasks such as the still face often restrict natural caregiving responses, that is, prohibiting touch or holding, which is known to be one of the most common caregiver responses to distress in everyday settings (Bornstein et al. 2017). As a result, these paradigms may provide an incomplete picture of caregiving in daily life and may underestimate the role of unrestricted, context‐dependent responses in supporting infants’ physiological regulation in real‐world settings.

Another key consequence of these standardized designs is that caregiver sensitivity, infant distress, and infant physiology are rarely captured all at the same time within a task phase or observation period. These designs therefore limit our ability to understand how caregiver behavior dynamically shapes infant physiological regulation during distress in real time. Given that RSA withdrawal is known to be rapid and dynamic (Porges 1995), this separation limits our ability to capture and understand how caregiver behavior, which varies in timing, quality, and appropriateness across distress interactions, shapes infant's physiological regulation in real time.

### 1.2 Capturing Infant Distress and Regulation in Real‐World Contexts

Ecologically valid recordings of caregiver responses to infant distress are critical for understanding the processes of infant emotion regulation in context. Recent work from our group has begun to leverage naturalistic recordings to better understand the real‐world, real‐time dynamics of parent–child distress interactions—specifically, how episodes of infant fussing and crying unfold moment‐to‐moment and how caregivers respond to and regulate that distress in the flow of everyday life. Using video observations of free‐flowing mother‐infant activity at home, we have shown that real‐time continuous measures of infant RSA can be time‐locked to the onset of free‐flowing events like infant crying or caregiver physical touch, allowing us to observe dynamic changes in parasympathetic activity to spontaneous distress and soothing behaviors in free‐flowing home interactions (Madden‐Rusnak et al., 2023, 2025). In these naturalistic works, we have found that infants display a significant decrease in RSA following the onset of unprompted crying and fussing (Madden‐Rusnak et al., 2023), and that caregiver holding following distress was associated with infants’ more adaptive RSA suppression following distress onset (Micheletti et al., 2025).

In addition, we have also used wearable audio devices to unobtrusively capture spontaneous episodes of infant distress in 24‐h home recordings without researchers present. Using these recordings, we annotated maternal sensitivity for each episode of infant distress, allowing us to observe how caregiving responses change across the day. On average, mothers—who responded to 78% of infant distress episodes on average—were relatively sensitive across the day, demonstrating mostly warm and/or otherwise appropriate responses to the level of their infants’ distress. We observed between‐persons differences in average sensitivity, consistent with prior literature (Bell and Ainsworth 1972; Hubbard and van Ijzendoorn 1991; Pianta et al. 1989). However, we also found substantial within‐mother variability, so much so that the distributions of the most and least sensitive mothers’ responses largely overlapped. These within‐mother variations were shaped by contextual factors. For example, mothers were less likely to respond sensitively when infant distress co‐occurred with other stressors, such as a toddler screaming in the background (de Barbaro & Madden‐Rusnak, 2026). These findings suggest that caregiver sensitivity is not a fixed trait but a dynamic behavior that fluctuates across time and context. This aligns with prior work showing that maternal sensitivity varies across caregiving domains (free play vs. mealtime; van Vliet et al. 2022), across daily routines (Teti et al. 2022), and as a function of the degrees of freedom or emotional tone of structured tasks (Bozicevic et al. 2025; Branger et al. 2019). Evidence from these studies highlights the importance of studying caregiver sensitivity as a context‐sensitive process that dynamically adapts to the situational demands of parenting.

Together, these findings underscore the importance of studying caregiving as it unfolds in daily life and suggest that both the timing and nature of caregiver responses are relevant to infant regulation. At the same time, they raise a critical next question: how does variation in the quality of caregiving responses across *and within* caregivers shape infants’ physiological regulation? One possibility is that infants’ physiological regulation primarily reflects stable differences between caregivers, such that infants of more sensitive caregivers consistently show more adaptive regulatory responses. Alternatively, regulation may operate as a dynamic, context‐sensitive process, such that infants show more adaptive physiological responses during moments when caregivers are more sensitive relative to their own typical behavior. Distinguishing between these possibilities is critical for understanding whether caregiver sensitivity functions as a stable characteristic, a moment‐to‐moment process, or both.

### 1.3 Moving Beyond Traits: Dynamic Regulation in the Context of Caregiver Support

Despite strong theoretical emphasis on regulation as a context‐sensitive process (Gross 2015; Lewis 2000; Porges 2011), empirical work has largely had to rely on isolated assessments of infant physiology where RSA is aggregated across task entire periods. This has led to regulation often being operationalized as a stable characteristic or profile. For example, prior studies have classified infants as “suppressors” or “non‐suppressors” based on exhibiting lower average RSA values in a single distress paradigm compared to the average RSA across a baseline or other non‐arousing task period (see review: Jones‐Mason et al. 2018). Such approaches have obscured potential within‐infant variability across repeated events and limit our ability to test whether physiological regulation tracks moment‐to‐moment changes in caregiver support.

While longitudinal assessments of RSA have shown moderate stability across infancy and childhood (Dollar et al. 2020; El‐Sheikh 2005; Morales et al. 2015), we know of no studies that have attempted to predict within‐infant variability in RSA across occasions at the same age. This limits our ability to observe how fluctuations in caregiver sensitivity might dynamically influence infant regulation in real‐time. Repeated observations across a day could reveal whether infants regulate more effectively during occasions when caregivers provide higher‐quality support, compared to episodes when the same caregiver provides less support. Wearable sensor paradigms offer a promising approach to unobtrusively capture repeated instances of real‐world behavior in context (Bolger and Laurenceau 2013; de Barbaro 2019; Harari et al. 2017). Critically, this allows researchers to move beyond static measures of sensitivity and regulation to test whether infant regulation dynamically adapts to momentary changes in caregiver sensitivity across the day or whether it is predicted by stable differences in caregivers’ overall sensitivity.

Recent methodological advancements have also made it possible to calculate continuous dynamic measures of RSA from continuous heart rate recordings, allowing researchers the ability to track physiological regulation during free‐flowing caregiver–infant interactions (Abney et al. 2021; Fisher et al. 2016; Gates et al. 2015). For example, home‐based semi‐structured observations that used continuous, high‐resolution measures of RSA found that larger fluctuations in infant RSA (greater standard deviations) within a free play task, predicted more behavioral problems as toddlers (namely, a higher composite of survey‐reported social competence and internalizing and externalizing behavioral symptoms) (Somers et al. 2021). These methods and technology can be extended, that is, synchronizing wearable audio recordings with wearable heart rate monitors over extended home recordings, making it possible to examine physiological regulation as a function of caregiver sensitivity across repeated episodes in everyday settings.

## 2 The Current Study

The current study investigates how variation in caregiver sensitivity to infant distress within‐ and between‐caregivers impacts infants’ real‐time regulation of distress in everyday settings. To do this, in a cross‐sectional sample of *N* = 23 caregivers and infants (ranging from 1 to 10 months of age), we leverage wearable sensors to capture continuous infant heart rate synchronized with full‐day home audio recordings, from which we annotate infant distress and caregiver sensitivity in response to each episode of distress.

Passive auditory sensing is perceived by families as a less intrusive method of capturing daily activity than using video cameras (Levin et al. 2021) while still preserving much of the context and moment‐to‐moment structure of infant distress and caregiver responses. In infancy, distress is communicated through vocal cues such as crying and fussing, and many caregiver responses to infant distress are also audible, meaning auditory recordings are well‐suited to capture these interactions. Caregivers are highly attuned to infant vocal signals, and hearing infant cries has been shown to activate extensive neurobiological systems involved in emotion processing, motivation, and action preparation (Joosen et al. 2013; Musser et al. 2012; Witteman et al. 2019). Maternal vocal behavior during infant distress events is not merely reactive but often functions as an active regulatory mechanism. Caregivers commonly use infant‐directed speech, soothing vocalizations, and prosodic modulation (“motherese”) to calm distressed infants. Contingently responsive vocal behavior has been shown to facilitate reductions in autonomic arousal and support emotion regulation (Kolacz et al. 2022; Provenzi et al. 2018; Trehub 2017), but see also Micheletti et al. (2025). Critically, as described above, in our own research group, auditory recordings have been successfully used to reliably capture the sensitivity of caregiver's responses to distress (de Barbaro & Madden‐Rusnak, 2026), including audible sounds associated with changes in caregivers’ physical proximity, approach, and contact with the infant (e.g., footsteps, changes in volume of voice, rustling of clothes during pick‐up).

Synchronizing audio recordings capturing these rich interactions in context with heart rate monitors allows us to explore infants' physiological responses to distress in unscripted, free‐flowing everyday home environments. We used time‐locked analyses to compare differences in infant RSA change between infants of caregivers who were more versus less sensitive on average across occasions of distress, as well as differences in infant RSA change between occasions of distress where caregivers were more sensitive than their own average versus less sensitive than their own average.

By examining differences in infant RSA change across individual differences in average caregiver sensitivity, as well as in response to variation in individual caregiver sensitivity across occasions, we can determine the extent to which caregiver sensitivity influences infant physiological regulation as a stable trait and/or a dynamic process.

Consistent with prior work, we hypothesize that infants of more sensitive caregivers will display adaptive vagal suppression following distress onset, whereas infants of less sensitive caregivers may show shallower or insignificant vagal suppression. Further, we also predicted that on occasions of distress that mothers respond more sensitively than their own average, infants will show stronger vagal suppression relative to occasions of distress when mothers are less sensitive than their own average.

Throughout the manuscript, we use the term caregiver when referring to the broader theoretical construct and maternal when specifically describing the primary caregiver participants in the current sample.

## 3 Method

### 3.1 Participants

Participants were 23 infants and their mothers from a larger cross‐sectional study using wearable audio and physiology sensors to capture up to a week of caregiver and infant behavior in the home. Infants and their mothers in the larger study were recruited via convenience sampling from the Austin, Texas metropolitan area. Advertisements for the study were posted to Facebook and the University of Texas at Austin event calendars and were also shared as fliers in local community health centers. Exclusion criteria included infants who were over 12 months of age, had congenital birth defects or a genetic condition, were multiple‐birth infants, or lived over 20 miles from the university. Data were collected between October 2017 and February 2020. The study design and data collection were approved by the University of Texas at Austin Institutional Review Board (Protocol #: 2017‐06‐0026).

From the original sample of *N* = 87 (infant age *M* = 4.3 months, SD = 2.4, Range = 0.8–10.4), 77 families shared continuous audio recordings (median duration = 70.51 h, SD = 36.02, Range: 1.06–180.40 h). More than half (63%, *n* = 55) of these families shared at least one continuous recording that lasted 21 h or more, which was used as the threshold of “full‐day recordings.” To ensure a broad range of infant ages in our data, families’ recordings were stratified across infant age and 38 were randomly selected for annotating infant distress and caregiver sensitivity. If selected families had more than one recording meeting the full‐day threshold, first, the longer of the recordings was selected, or otherwise, recordings were randomly selected if they were of similar length. From this sample of 38 families’ recordings, one dyad's recordings were excluded for having only two infant distress events, as our inclusion criteria threshold was at least three instances of infant distress. From the 37 families with sufficient infant distress data, another 14 were excluded due to poor‐quality heart rate data or having heart rate data that did not align with audio recordings (further details on criteria for determining physiological quality are detailed in Section 3.5 below).

The final sample included 23 infants (Mean age = 4.71 months, SD = 2.37 months; Range: 0.87–10.40 months) and their mothers (Mean age = 29.73 years, SD = 4.88 years; Range: 22–39 years). In this sample, 11 (47.83%) of infants were female. Infant race/ethnicity from this final sample was 60.87% White/Non‐Hispanic, 17.39% Hispanic/Latino, 17.39% Two or More Races, and 4.35% White/Hispanic. Approximately 52.174% of families had a household income between $50,000 and $99,999, 34.783% with an income above $99,000, and 13.04% below $50,000. Approximately 78.26% of mothers attended some college or earned a college degree. Most mothers were employed outside of the home (56.52%), and 91.30% of dyads in this subsample indicated that English was the primary language spoken at home. Most mothers were married (91.30%). Of the households that reported the total number of children in the home (52%), most had another child in the home (67%). See Table 1 for complete sample characteristics.

**TABLE 1:** Sample characteristics (*n* = 23).

		*M* (SD), Range or *N* (%)
Infant		
	Age, months	4.71 (2.37), 0.87–10.40
	Gestational age, weeks	38 (2.59), 31–41
	Sex, female	11 (47.83%)
	Race/ethnicity	
	White, Non‐Hispanic	14 (60.87%)
	Hispanic/Latinx	4 (17.39%)
	White, Hispanic	1 (4.35%)
	Two or more races	3 (17.39%)
Mother		
	Age, months	29.73 years (4.88), 22–39 years
	Race/Ethnicity	
	White, Non‐Hispanic	16 (69.57%)
	Hispanic/Latina	4 (17.39%)
	White, Hispanic	1 (4.35%)
	Two or more races	2 (8.69%)
	Education	
	High school diploma	3 (13.04%)
	Some college	2 (8.70%)
	College degree	8 (28.57%)
	Graduate school degree	11 (39.29%)
	Family status	
	Married	21 (91.30%)
	Living with partner, not married	2 (8.70%)
	Household income	
	Under $25k	1 (4.35%)
	$25k–$49k	2 (8.70%)
	$50k–$74k	6 (26.09%)
	$75k–$99k	6 (26.09%)
	Over $100k	8 (34.77%)
	Primary household language	
	English	21 (91.30%)
	Spanish	2 (8.70%)
	Maternal work status	
	Not employed outside the home	10 (43.48%)
	Employed full‐time	7 (30.43%)
	Employed part‐time	6 (26.09%)

### 3.2 Procedure: Home Recording Session

Mothers were requested to have their infants wear a suite of wearable sensor devices for one 48‐h and two 24‐h shifts over the course of a week, for 72 total h of home recording data. Prior to home recording sessions, trained research assistants traveled to participants homes to complete a 1‐h introductory session where written and informed consent was obtained. Mothers were taught how to place the wearable heart rate monitor on the infant and the placement of the audio recording device that infants would wear in a custom vest (details below). Participants were told to remove devices during bathing but were otherwise encouraged to wear devices continuously throughout everyday activities across their chosen shifts. Recording “shifts” across the week were chosen by mothers during periods when they were both with their infants and primarily responsible for their infant's caregiving. To increase compliance, text reminders were sent throughout the week. At the end of a recording week, research assistants collected sensors and provided up to $100 in compensation along with a small toy.

### 3.3 Infant‐Worn Audio Recordings (LENA)

Infant‐worn audio recordings were collected during home recording sessions using the Language ENvironment Analysis (LENA) system. LENA is a small, child‐safe audio recording device widely used in early language research, typically worn in a front pocket of a custom infant vest or T‐shirt. The device continuously captures the auditory environment of the child, including sounds within approximately 6–10 feet of the child. Although standard recording capacity is approximately 16 h, extended 24‐h recordings were enabled in collaboration with LENA. Caregivers were instructed on how to pause or stop recordings at any time and could request deletion of recordings without restriction.

### 3.4 Measures

#### 3.4.1 Audio Data Annotation

For each of our 38 dyads, a full‐day home audio recording was annotated using a two‐stage process. First, all instances of infant distress were identified and temporally bounded. Second, a separate team of annotators coded caregiver sensitivity for each identified distress episode (see below).

All annotations were conducted using the free software ELAN, which allows for alignment of multiple tiers of isolated or parallel coding across video or audio data (https://archive.mpi.nl/tla/elan). To reduce coder fatigue and maintain consistency, recordings were segmented into continuous 6‐h blocks, with annotators typically working in 1‐ to 2‐h sessions. Coding was based exclusively on audible information. Annotators used vocal and contextual acoustic cues (e.g., infant vocal intensity, caregiver vocalizations, movement‐related sounds such as clothing rustling or changes in volume to indicate shifts in proximity) to infer infant state and caregiver behavior. Although caregivers were not visually identified, annotators were assigned extended continuous segments, allowing them to reliably distinguish caregivers based on their vocal characteristics and interaction patterns. Multiple annotators contributed to most recordings; however, the number of annotators per participant was not associated with mean caregiver sensitivity (t = 0.76, *p* = 0.452) or within‐person variability (t = 0.57, *p* = 0.572).

##### 3.4.1.1 Infant Distress Annotations

For a majority of participants recordings (32 of 38), instances of infant distress were annotated by research assistants. In six additional recordings, distress was first annotated by a validated algorithm and then verified by human raters. This effort was completed to increase sample size and improve generalizability of our results.

###### 3.4.1.1.1 Human Annotation

For 32 of 38 recordings (19 out of 23 in the current study), all distress vocalizations were manually annotated by trained RAs. Distress included fussing or crying. Following existing coding schemes (Hubbard and van Ijzendoorn 1991), cry vocalizations were required to have a minimum duration of 3 s and were merged if separated by less than 5 s. Fussing did not have a minimum duration. Distress vocalizations were collapsed into the singular category of distress for analysis.

Reliability between annotators was assessed across 12 continuous 6‐h recordings (72 h total) that were double‐coded by all annotators. As annotations included precise onset and offset timing, reliability was assessed using Cohen's kappa (Bakeman and Gottman 1997), yielding *κ* = 0.85, indicating strong agreement.

###### 3.4.1.1.2 Algorithm‐Derived Annotations

For six recordings (four included in the current study), distress was initially identified using an algorithm developed by our research group (Yao et al., 2022). This model combines acoustic features from the raw audio (e.g., signal energy, spectral structure, and mel‐frequency cepstral characteristics derived from the audio signal) with deep spectrum features extracted from a convolutional neural network (modified AlexNet architecture) classified using a support‐vector machine. The model generated frame‐level binary classifications (“distress” vs. “not distress”) at a fine temporal resolution.

The model was trained and validated on annotated naturalistic infant audio using leave‐one‐participant‐out cross‐validation and evaluated on continuous 24‐h recordings to reflect real‐world performance (Yao et al., 2022). Additional validation studies (Micheletti et al., 2023) demonstrated that our algorithm achieved substantially higher agreement and accuracy with human coders (*κ* = 0.53; ∼90%–95% agreement, F1 = 0.53) compared to the built‐in LENA detection algorithm (*κ* = 0.19, F1 = 0.17).

Importantly, all algorithm‐identified segments in the present study were subsequently reviewed during caregiver sensitivity coding, and annotators confirmed that each segment contained true infant distress or was otherwise excluded from coding. During this process, annotators also verified that vocalizations originated from the focal infant—rather than siblings or other children—based on contextual and acoustic cues (e.g., proximity to the recording device, caregiver responses directed toward the infant, and co‐occurring handling sounds). Thus, all distress episodes included in analyses were ultimately human‐verified and confirmed as originating from the focal infant, regardless of initial detection method.

###### 3.4.1.1.3 Derivation of Infant Distress Episodes

Individual distress vocalizations occurring within 5 min of one another were collapsed into a single “distress episode.” This approach aligns with prior work conceptualizing caregiver sensitivity as a global response to a bounded distress interaction and facilitates comparison with laboratory‐based paradigms that assess sensitivity across brief interaction windows.

##### 3.4.1.2 Caregiver Sensitivity Annotations

Caregiver sensitivity was coded for each distress episode by a second team of annotators. Caregiving sensitivity ratings were informed by established observational coding frameworks. Specifically, the 7‐point global sensitivity scales used in the present study (7 = highly sensitive, 1 = highly insensitive) are conceptually derived from the NICHD Early Child Care Research Network coding system and subsequent adaptation for sensitivity to distress contexts (Leerkes et al. 2024).

Following Leerkes’ guidelines and in alignment with Ainsworth's (1969) framework, ratings reflected three core dimensions: contingency (timing of responses relative to distress onset), appropriateness (the match between caregiver response and the intensity and nature of infant distress), and affective quality (e.g., vocal warmth, soothing prosody). Coders relied on auditory and contextual cues (e.g., vocal tone, intensity, and changes in proximity or handling sounds) to make these judgments. Rather than applying a fixed latency threshold, contingency was evaluated relative to the unfolding distress episode, integrating response timing with the proportion and appropriateness of responses.

Appropriateness was evaluated in reference to the intensity of infant distress vocalizations. For example, neutral vocalizations or shushing in response to intense crying were considered mismatched and received lower ratings, whereas similar responses to mild fussing could be considered appropriate. Sensitivity was coded globally across all responses within the episode, including those from one or multiple caregivers, allowing us to capture the overall sensitivity experienced by the infant rather than attributing behavior to a specific individual. It should be noted that, in related work using the same dataset (*N* = 38), the majority of distress episodes were responded to by mothers (78%), most often by mothers alone (71.5%), and less frequently by other caregivers, which were more commonly the father, followed by other adult family members. A smaller proportion of episodes had no caregiver response (15.6%). See de Barbaro and Madden‐Rusnak (2026) for additional detail on distress episodes and contextual coding.

Interrater reliability for caregiver sensitivity was assessed across a subset of 13 six‐hour recording segments from distinct families (88 distress episodes within 78 h of audio). As caregiver sensitivity ratings were numerical judgments, we used intra‐class correlations (Type; 1,3,) (Shrout and Fleiss 1979). Coders achieved a moderate reliability score (ICC = 0.70), similar to other recently published maternal sensitivity annotation studies (e.g., Leerkes et al. 2009, ICCs ranging from 0.69 to 0.80; Woodhouse et al. 2020, ICCs ranging from 0.69 to 0.98).

### 3.5 Ambulatory Heart Rate Monitoring

Infant heart rate data were obtained using wireless Movisens Move4 Acceleration/ECG chest sensors (Movisens GmbH, Karlsruhe, Germany), recorded at a sampling rate of 1024 Hz. Infants wore the sensor in the center of their chests on the nipple line using hypoallergenic, gentle‐adhesive, foam gel pediatric electrodes.

To ensure data validity, heart rate data were only included if Movisens output indicated the sensor was on and the infant was not asleep. Infant R‐R intervals were extracted using Movisens DataAnalyzer (Movisens GmbH, Karlsruhe, Germany). The high‐frequency bandpass filter was set to an infant‐specific range of 0.24–1.04 Hz (Porges 1988). Interbeat intervals (IBIs) were then extracted offline using ARTiiFACT (Kaufmann et al. 2011). To clean IBI data, first, Kubios HRV software was used (Kubios Oy, Kuopio, Finland, v. 3.4.3) to remove artifacts across each of the full‐day recordings for all participants. The “very low” threshold cleaning was used, meaning IBIs that were larger or smaller than the local average within a 0.45‐s window were marked for correction and then removed. This threshold of “very low” was selected following visual inspection of corrections, as it removed clear outliers while leaving the natural variability in IBIs untouched. Corrections were then made by replacing the identified artifact with interpolated values using a cubic spline interpolation.

The average beats corrected in Kubios were 1.88% (Range: 0.01%–5.45%). Following automatic beat correction, beats were then replaced if they were greater than or equal to 200 or less than 20 beats per minute with a missing value indicator (NaNs) in MATLAB. Missing values were replaced using interpolation with a cubic spline. The average beats deleted, that is, replaced with NaNs across full‐day ECG recordings, was 0.02% (Range: 0%–0.16%).

Of the 14 infants removed from the original 38 for ECG quality reasons, three were excluded because they had no ECG data that overlapped with audio recordings, while 11 were excluded because their ECG data had a combined percentage of beats corrected and deleted greater than 10% across either their full‐day recording or across segments that aligned with infant distress episodes and were excluded from the current analyses.

The analytic sample (with usable RSA data) did not differ from excluded cases, including the one infant excluded for having less than three distress episodes, on key demographic variables, including age, gender, race, or family income (all *p* > 0.45), suggesting minimal risk of selection bias.

For greater details on ECG signals and data cleaning practices, refer to the resource by Kligfield et al. (2007). Artifact and beats deletion thresholds were inspired by prior works by Dollar et al. (2020) and the detailed resource by Clifford et al. (2006).

### 3.6 Dynamic RSA Timeseries

Dynamic RSA timeseries were calculated in MATLAB using the technique openly shared and validated by Abney et al. (2021). This technique expands the Porges and Bohrer (1990) method to create a continuous measure of RSA. First, cleaned IBI time series were resampled to 5 Hz, then beats‐per‐minute and RSA were calculated and filtered using child‐normed parameters (0.30–1.30 Hz). Continuous RSA was then calculated using a 15‐s sliding window (updated every 200 ms). This sliding window size was determined to have the greatest stability at the highest time resolution. For more details on this process, we encourage readers to enjoy the full method text by Abney et al. (2021).

## 4 Data Analysis

### 4.1 Calculating Time‐Locked Changes in Infant RSA

We used time‐locked event‐related analyses to observe dynamic changes in RSA relative to infant distress episode onsets. To do so, we used onset times from the annotated distress episodes to extract and align RSA timeseries segments beginning at 0.2 s prior to each episode onset and ending 60 s after onset. Episode onsets were represented at time = 0.

Next, for each aligned segment, we calculated relative change in RSA by subtracting all the RSA values within a segment from the baseline RSA value preceding onset (*t* = −1). These values, in the resulting timeseries, are centered such that 0 reflects RSA at baseline (i.e., just prior to distress onset). Negative values indicate decreases in RSA relative to baseline (consistent with vagal suppression and increased sympathetic arousal), whereas positive values indicate increases in RSA (greater parasympathetic activation).

We then averaged these time‐locked segments across all qualifying episodes to generate a mean RSA change time series for each analysis (described further below). We also computed the average RSA change across the full 60‐s window by taking the mean of the time‐locked change values within each segment.

Durations of infant distress episodes were highly right‐skewed (Range: 1 s–58 min; median = 6 min, 6 s). Because episodes could include multiple vocalizations grouped within a 5‐min window, we focused on the first 60 s following episode onset to capture a period with the highest likelihood of active distress across episodes. This window also aligns with our prior work demonstrating that changes in infant RSA in response to distress are most pronounced within the first 30 s following onset (Madden‐Rusnak et al., 2023).

### 4.2 Caregiver Sensitivity Analyses

To determine the effects of within‐ and between‐participant differences on infant regulation, we created two groupings of high‐ and low‐sensitivity distress episodes.

#### 4.2.1 Between‐Subjects Comparisons

Our between‐subjects comparison groups were made by dividing each *participant* into one of two groups: mothers with above‐average sensitivity and mothers with below‐average sensitivity. Groups were created using the sample average sensitivity rating of 4.85, resulting in *n* = 14 dyads in our above‐average sensitivity group, with a total of *n* = 199 distress episodes, and *n* = 9 dyads in our below‐average sensitivity group, with a total of *n* = 87 distress episodes across all mothers. To compare changes in RSA following infant distress onset between high‐ and low‐sensitivity mothers, we averaged time‐locked RSA timeseries for all distress episodes identified within recordings from high‐sensitivity mothers (*n* = 199 episodes) and low‐sensitivity mothers (*n* = 87 episodes). This resulted in two timeseries representing the average time‐locked changes in infant RSA, one for mothers with above‐average sensitivity and one for mothers with below‐average sensitivity.

#### 4.2.2 Within‐Subjects Comparisons

To determine the effects of within‐person fluctuations in maternal sensitivity on infant regulation in real time, we split each distress *episode* into one of two groups: episodes in which the mother was “more sensitive than her own average” and episodes in which the mother was “less sensitive than her own average.” To do so, for each mother, we calculated an average sensitivity score, defining all episodes with a score above the mother's own average as occasions that were “more sensitive than mother's average” and all episodes with a score below the mother's own average as occasions that were “less sensitive than mother's average.” Values equal to the mean were grouped into the below‐mean category. This resulted in *n* = 136 distress episodes in which mothers were more sensitive than their own average and *n* = 150 events where they were less sensitive than their own average. To compare changes in RSA following infant distress onset on occasions when mothers were more sensitive than usual versus less sensitive than usual, we averaged time‐locked RSA timeseries for all “more sensitive occasion” episodes (*n* = 136) and “less sensitive occasion” episodes (*n* = 150). This resulted in two timeseries representing the average time‐locked changes in infant RSA for occasions when mothers were more sensitive than usual versus occasions when mothers were less sensitive than usual.

### 4.3 Physiological Significance Testing

Consistent with prior works using continuous RSA time‐locked to naturalistic events (Madden‐Rusnak et al., 2023, Abney et al. 2021), we assessed whether changes in RSA differed significantly from the pre‐distress baseline. Because RSA values were centered such that 0 represents the value immediately prior to distress onset, significance testing evaluated whether RSA change values deviated from zero over time.

We computed 95% confidence intervals (CIs) for the mean RSA change time series at each time point. Time points were considered significantly different from baseline when the CI did not include zero (i.e., fell entirely above or below baseline levels). If the CI did not fall below zero at any point, this was interpreted as a lack of significant vagal suppression following distress onset.

To reduce spurious detections due to noise and multiple comparisons, we imposed a temporal contiguity criterion: a change was only classified as significant if the CI excluded zero (no change) for at least three consecutive seconds (15 samples). Shorter deviations were not considered reliable. Additionally, gaps of less than 3 s between significant segments were merged into a single continuous period of change. This approach ensures that identified effects reflect sustained physiological responses rather than transient fluctuations.

## 5 Results

**FIGURE 1:**
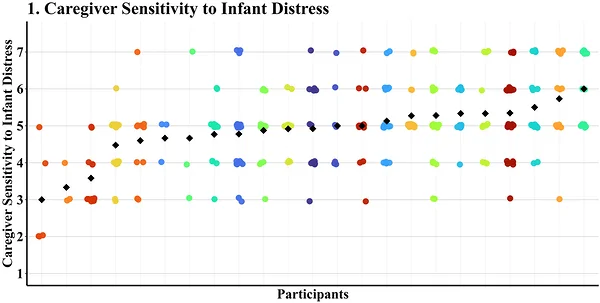
Caregiver sensitivity to infant distress across dyads. Each column represents a participant (dyad), ordered by their mean caregiver sensitivity across all distress episodes. Colored points represent individual episodes of infant distress, with vertical position indicating caregiver sensitivity (points are jittered for visibility). Black diamonds indicate each caregiver's mean sensitivity score. For between‐participant analyses, dyads were classified as above‐ or below‐average based on the sample mean sensitivity score (*M* = 4.85). For within‐participant analyses, episodes were classified relative to each caregiver's own mean sensitivity, with episodes above the caregiver‐specific mean categorized as higher‐sensitivity occasions and those below as lower‐sensitivity occasions.

Our final sample of *N* = 23 infants averaged 14.34 distress episodes (Range: 3–27) in each 24 h recording, totaling 332 distress episodes; of these, 75 (22.59%) from four infants were initially identified by our cry algorithm and later validated by human coders prior to sensitivity annotation. Distress episode durations ranged from 1 s to nearly 58 min, with a strong right skew. The 25th percentile of durations was 31.4 s, the median was 169.1 s (approximately 2.8 min), and the 75th percentile was 531.4 s (just under 9 min). The average sensitivity across our dyads average sensitivity scores was 4.85 (SD = 0.72), where a score of five corresponds to responses that are predominantly responsive, but some responses may be somewhat delayed, or some milder fusses may be ignored. See Figure 1 for a display of all sensitivity ratings.

### 5.1 Infant Vagal Suppression Is Moderated by Average Caregiver Sensitivity

**FIGURE 2:**
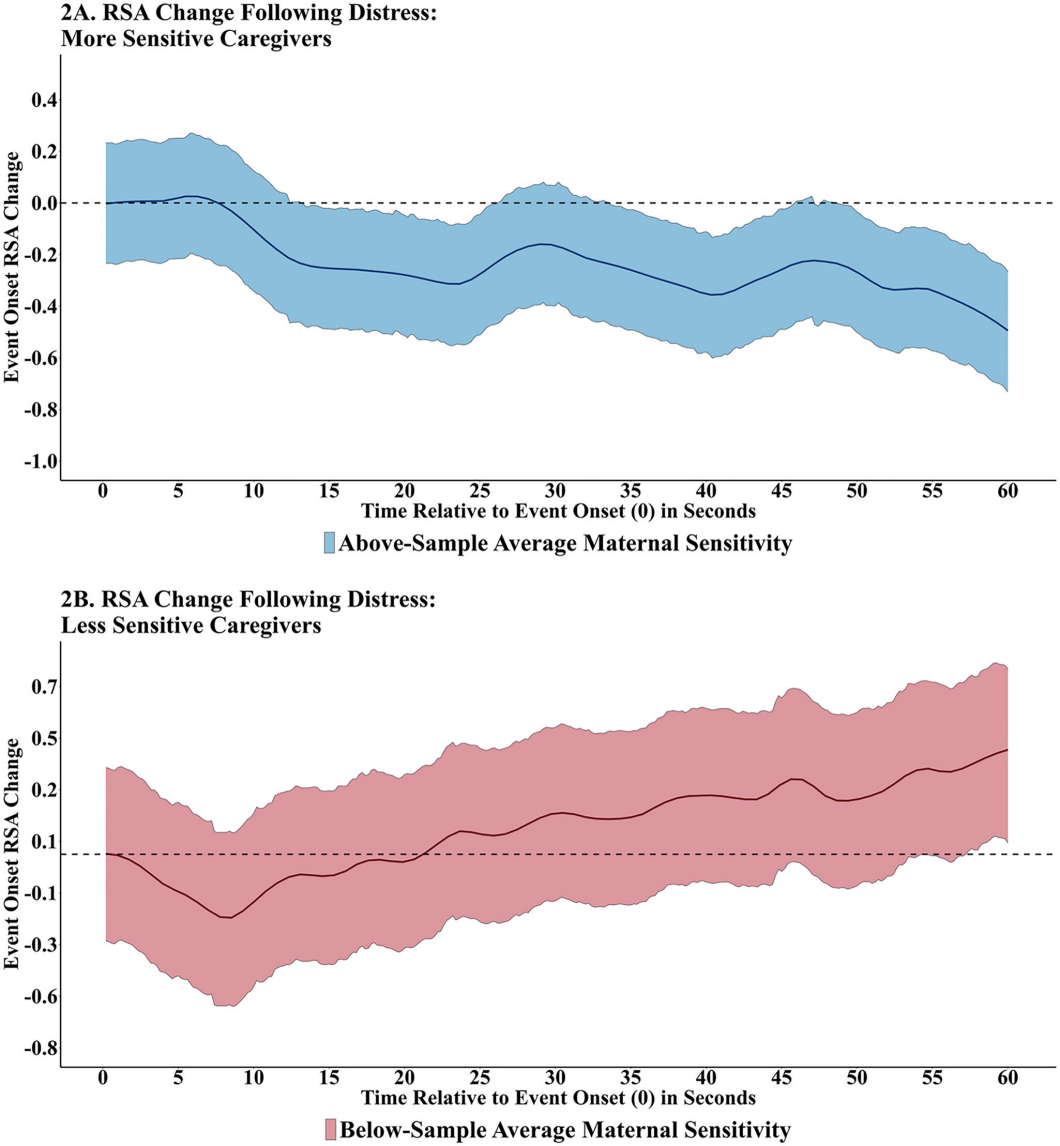
Infant RSA change following distress onset: Between‐participants analyses examining more versus less sensitive caregivers. (A) Average infant RSA change following distress episode onset for infants of more sensitive caregivers (*n* = 199 episodes of distress from *n* = 14 dyads). (B) Average infant RSA change following distress episode onset for infants of less sensitive caregivers (*n* = 87 episodes from *n* = 9 dyads). Time = 0 represents onset of distress episode. *Y* = 0 represents RSA value prior to onset of infant distress.

Infants of more sensitive caregivers showed significant vagal withdrawal following infant distress onset. By contrast, infants of mothers who were less sensitive than the average caregiver in our sample showed no evidence for vagal suppression. The upper 95% CI for infants of more sensitive caregivers fell below *Y* = 0 (pre‐onset value) from 12.4 to 26.2 s post‐onset and again from 33.4 to the end of our data inclusion of 60 s (*p* <  0.05), indicating a statistically significant decrease at those intervals; see Figure 2A. There was no period of time in which infants of less sensitive caregivers showed changes in RSA that were significantly different from prior to onset beyond our significance threshold; see Figure 2B.

The average change in RSA for infants of more sensitive caregivers across our inclusion period of 60 s was −0.23 (SD = 0.12) and 0.10 (SD = 0.17) for infants of less sensitive caregivers.

### 5.2 Infant Vagal Modulation Is Dynamically Impacted by Within‐Person Changes in Caregiver Sensitivity

Our within‐group analysis revealed that infants showed significant vagal suppression only on occasions when mothers were more sensitive than their own average but not on occasions when mothers were less sensitive than their own average. The decrease in infant RSA following distress was significantly below zero from 9.4 to 27.8 s post‐distress onset (*p* < 0.05), evidenced by the 95% CI falling below zero at those times; see Figure 3.3A.

**FIGURE 3:**
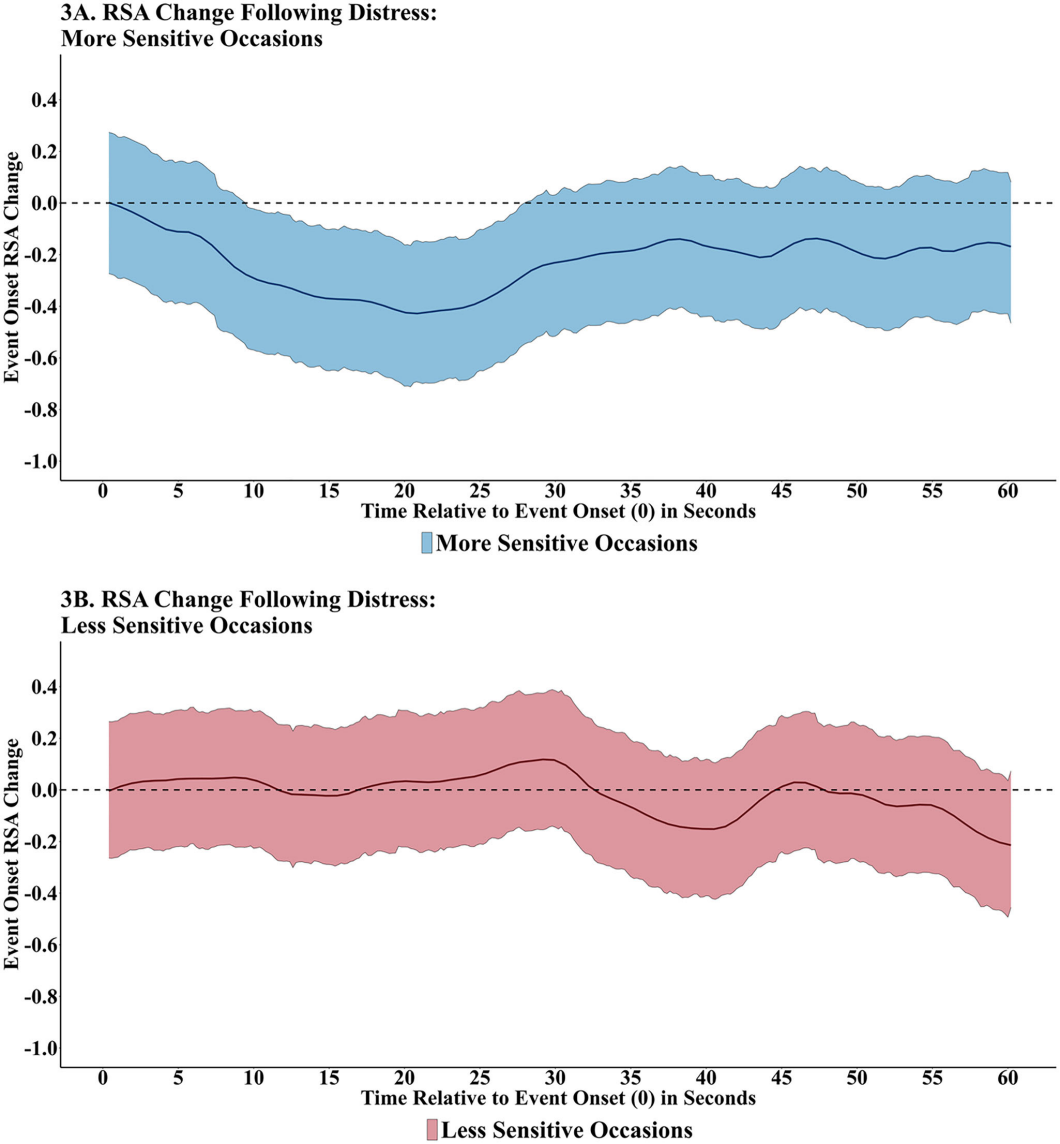
Infant RSA change following distress onset: Within‐participant analyses examining more versus less sensitive occasions. (A) Average infant RSA change following distress episode onset for occasions on which mothers responded more sensitively than their own average. (*n* = 136 episodes, drawn from *n* = 23 dyads). (B) Average infant RSA change following distress episode onset for occasions on which mothers responded less sensitively than their own average (*n* = 150 episodes, drawn from *n* = 23 dyads). Time = 0 represents onset of distress episode. *Y* = 0 represents RSA value prior to onset of infant distress.

Average change in RSA for infants on occasions when their caregiver was more sensitive was −0.23 (SD = 0.11) and −0.02. (SD = 0.08) for infants on occasions when their caregiver was less sensitive than their own average.

## 6 Discussion

Using a novel, naturalistic approach that synchronized wearable audio recorders with heart rate sensors, we captured real‐time changes in infants' physiology in response to ecologically valid variation in maternal sensitivity over the course of a day. Infants of mothers with above‐average sensitivity showed adaptive vagal suppression following distress, an indicator of effective physiological regulation, as did infants on occasions that their mothers were more sensitive than their own average. By contrast, neither mothers with below‐average sensitivity nor less‐than‐average sensitive occasions were associated with adaptive infant vagal suppression following distress onset. These findings suggest that infant physiological regulation is shaped by both stable, between‐mother differences in sensitivity and dynamic, within‐mother fluctuations in sensitivity across the day. To our knowledge, this is the first study to capture infants' real‐time physiological responses to spontaneous ecologically valid changes in maternal sensitivity in everyday home environments. Beyond our empirical contributions, this work demonstrates the feasibility of capturing synchronized caregiver–infant behavior and physiology in daily life, offering a powerful tool for advancing our understanding of how everyday interactions influence infant regulation.

An important question is how these findings relate to prior work conducted in structured laboratory paradigms. Although we hesitate to provide direct comparisons of effect sizes without more systematic investigations of comparativeness between different RSA measurement and analytic approaches, the overall pattern of our results closely mirrors those observed in controlled settings. Specifically, laboratory studies using paradigms such as the still‐face have consistently found that greater caregiver sensitivity, particularly during periods of recovery, that is, the reunion phase, is associated with more adaptive physiological regulation, including patterns of parasympathetic engagement and baseline RSA level recovery (e.g., Conradt and Ablow 2010; Moore et al. 2009). The present findings extend works like this by demonstrating a similar pattern in naturalistic environments, where caregiving unfolds amid substantially greater contextual variability (Branger et al. 2019; de Barbaro and Madden‐Rusnak 2026; van Vliet et al. 2022). This convergence suggests that the association between caregiver sensitivity and infant physiological regulation may reflect a robust, generalizable process rather than one that is specific to structured laboratory conditions. At the same time, our results also expand the literature by showing that these associations operate not only at the level of stable differences between caregivers, but also through moment‐to‐moment fluctuations in sensitivity within the same caregiver–infant dyad. Prior work has often classified infants into physiological profiles (e.g., “suppressors” vs. “non‐suppressors”) based on vagal suppression observed in a single distress task (Jones‐Mason et al. 2018). While our findings provide some support for this framework at the between‐person level, they also reveal important nuances. Specifically, we observed substantial within‐infant variability, such that the same infant exhibited different regulatory responses depending on the sensitivity of caregiver support during a given episode. This pattern suggests that trait‐based categorizations may oversimplify infant regulation by failing to capture its dynamic, context‐dependent nature.

By capturing repeated, real‐world interactions across the day, we show that infants who showed “non‐suppressor profiles” when averaging across all interactions with a less‐than‐average sensitivity caregiver also showed adaptive RSA suppression on occasions when their caregiver was more sensitive than their own average. This suggests that infant regulation is not fixed, but dynamically responsive to real‐time variation in caregiver support. Our findings are also consistent with theoretical frameworks that posit that emotional regulation is a dynamic process sensitive to factors including the context and level of support available (Gross 2015; Lewis 2000; Porges 2011). It is also consistent with prior work that has shown that infants’ stress responses are sensitive to context. For example, infants exhibit higher levels of the stress hormone cortisol when they interact with a less sensitive caregiver, relative to a more sensitive caregiver, when their mother is absent (Gunnar et al. 1992).

Our findings also build on prior work demonstrating that maternal sensitivity is highly variable within individuals across the day (de Barbaro & Madden‐Rusnak, 2026, see also Figure 1). While attachment theory emphasizes the consistency of maternal sensitivity for infant attachment development, our work suggests that variability in maternal sensitivity at home is normative and meaningful. Our current findings add to these results by demonstrating that both between‐mother differences and within‐mother fluctuations are linked to infant physiological regulation. Our results suggest that infants are responsive to dynamic changes—increases as well as decreases—in maternal sensitivity, while also reflecting the overall quality of caregiving they experience. This insight may help reframe caregiving for parents who feel pressure to be perfectly consistent, highlighting that meaningful improvements in sensitivity, even in individual moments, can have measurable positive effects on their infants’ regulation.

Across analyses, vagal suppression emerged within approximately 10–15 s following distress onset and persisted for sustained intervals when caregivers responded sensitively. This pattern is consistent with models of autonomic flexibility, which emphasize rapid parasympathetic withdrawal as a mechanism supporting adaptive engagement with stress (Porges 1995). In contrast, the absence of sustained suppression in lower‐sensitivity contexts suggests not simply a delay in response, but a qualitatively different profile of vagal activity. These findings highlight the importance of examining the timing and duration of physiological responses, not just their presence or absence. Naturalistic, time‐locked approaches are uniquely positioned to capture these fine‐grained dynamics.

Our results have several applied implications. First, they suggest that caregivers may not need to achieve uniformly high levels of sensitivity to effectively regulate their infants. Mothers with higher average sensitivity showed better regulation on average, but occasions of higher‐than‐average sensitivity also had significant benefits for infant physiological regulation. This aligns with “good enough” caregiving frameworks and may help alleviate unrealistic expectations of perfect consistency (Winnicott 1976). Second, the use of passive sensing methods offers a scalable and less intrusive way to assess caregiving in real‐world settings, with potential applications for early identification of risk and monitoring of intervention effects (de Barbaro 2019; Levin et al. 2021; Li et al. 2025). Finally, these approaches may help identify contexts in which caregivers are more likely to struggle with sensitivity, providing opportunities for targeted, context‐specific support.

Future research should aim to specify how everyday caregiver responses to distress and corresponding infant physiological regulation profiles predict longitudinal outcomes, including secure attachment and emotion regulation. Longitudinal designs that integrate repeated measures of naturalistic interactions will be particularly valuable for identifying what constitutes a “typical” or developmentally supportive range of caregiving. For example, given that even the most sensitive caregivers in our sample occasionally responded insensitively (e.g., scores of four or below), an important question is what frequencies, proportions, or intensities of such variability are associated with later secure versus insecure attachment. Addressing these questions may help provide caregivers with a more balanced and achievable perspective on parenting.

A related and critical direction is to directly compare the predictive validity of sensitivity assessed in naturalistic versus laboratory contexts. Laboratory paradigms are designed to isolate regulatory processes under controlled conditions, whereas naturalistic assessments capture caregiving as it unfolds amid competing demands and environmental variability, and there is precedence for significant changes in behavior between these settings (Abels et al. 2017; Brzozowska et al. 2021; Dahl 2017). Regardless, approaches likely index distinct but complementary constructs. Laboratory‐based assessments of sensitivity may reflect caregivers’ capacity to regulate and respond to infant cues in structured, supportive environments, offering insight into caregiving skills that could be strengthened through behavioral intervention. By comparison, assessments conducted in everyday settings may better characterize how caregiving is implemented amid the competing demands and constraints of daily life, illuminating contexts in which families may benefit from additional structural support. Longitudinal studies that examine both approaches within the same sample will be essential for determining which better predicts downstream developmental outcomes, or whether their combination yields the strongest predictive utility.

### 6.1 Potential Limitations

First, a substantial portion of infants (14 of 38; 37%) did not have usable physiological data and were excluded from our analyses. This reflects the inherent challenges of collecting high‐quality ambulatory physiological data in naturalistic settings. Although our analytic sample did not differ from excluded participants on key demographic characteristics, data loss may still introduce bias if missingness is associated with factors such as infant movement or distress. Future studies could enhance the robustness of findings by incorporating repeated data collection sessions, selecting participants to annotate based on availability of high‐quality physiological data, or using higher quality monitors. Each of these techniques has its challenges: collecting more data is a higher burden and more expensive, selecting based on data quality could lead to biases if, for example, data quality is associated with motion or negativity, and higher quality monitors (e.g., Mindware or Biopac) are larger and more expensive. However, protocols to increase the volume of usable data will be important to expand sample sizes and the breadth of observations in future studies.

Second, caregiver sensitivity was coded using audio‐only recordings. While this approach enables scalable, low‐burden data collection in naturalistic environments, it limits access to non‐audible dimensions of caregiving, such as mutual gaze or some types of physical soothing (e.g., holding, stroking). As a result, our measure may underestimate certain dimensions of sensitivity or differentially weight auditory cues. Future studies integrating multimodal sensing (e.g., audio combined with wearable proximity or movement sensors) could provide a more comprehensive account of caregiving behavior while preserving ecological validity.

Third, our analyses did not account for the specific causes of infant distress, such as biological needs (e.g., changing or feeding) versus stressful perturbations (e.g., sibling interactions or lack of attention), nor levels of distress (fussing vs. crying). Future research should consider these contextual factors, as behavioral and physiological responses may vary depending on the cause or intensity of distress. Incorporating more fine‐grained distinctions in distress type and intensity will be important for understanding how sensitivity operates across varying caregiving demands.

Fourth, the relatively wide infant age range in our study introduces developmental heterogeneity that was not explored in the current work. Infant distress signaling, regulatory capacity, and caregiver responsiveness can change substantially across early infancy. For example, longitudinal work suggests that infant negative emotionality and mother–infant interaction patterns vary across the first 6 months of life based on infant temperament profiles (van den Bloom & Hoeksma 1994), while studies of reactivity and regulation indicate that regulatory behaviors become more differentiated by later infancy (Braungart‐Rieker & Stifter 1996). A recent systematic review further shows that associations between distress expression and regulation vary by age and regulatory strategy, with substantial heterogeneity across studies (Gennis et al. 2022). Although our design prioritized ecological validity by sampling naturally occurring distress across a broad developmental window, future studies with larger samples should explicitly model infant age as a moderator of RSA reactivity.

Fifth, the relatively wide infant age range (1–10 months) introduces meaningful developmental heterogeneity. Based on prior literature, it is known that both infant distress signaling and caregiver responsiveness undergo substantial changes across this period. For example, while distress frequency does not change much from 1 month old to 9 months, the duration of distress has been shown to decrease by half (Hubbard & van Ijzendoorn 1991). Similarly, baseline RSA levels and patterns of vagal withdrawal are known to evolve across early infancy and throughout early childhood (Dollar et al. 2020; El‐Sheikh 2005; Jewell et al. 2018). Although our design prioritizes ecological validity and captures regulation across a broad developmental window, future studies with larger samples should explicitly model age as a moderator to determine whether the observed associations vary across developmental stages or reflect more generalizable regulatory processes.

Lastly, our study did not account for maternal psychological characteristics, which have been shown to influence caregiver–infant co‐regulation (Goodman 2007; Ostlund et al. 2017). It is possible that even when caregivers are behaviorally sensitive, psychological distress may limit the effectiveness of their support, perhaps due to physiological dysregulation or otherwise reduced engagement. Similarly, infants may vary in their capacity to benefit from sensitive caregiving based on regulatory flexibility or stress reactivity (de Barbaro et al. 2024). Future work should incorporate maternal mental health, physiological reactivity, and infant temperament measures to better understand how individual differences may moderate the effects of moment‐to‐moment caregiving on infant physiological regulation.

## 7 Conclusion

At home, caregivers respond to countless cries. Sometimes infants cry when a caregiver changes an infant's diaper, other times, when they are cooking or responding to a sibling's tantrum. The everyday contexts of infant distress can affect caregiver sensitivity across the day, with implications for infant regulation. By examining repeated episodes of spontaneous distress occurring throughout the day, we found that infants more actively regulate distress physiologically with caregivers who are more sensitive than average, and on occasions when their caregivers provide more sensitive responses than average. Overall, infant's physiological regulation is influenced by their caregiver's typical level of sensitivity while also adapting dynamically to changes in sensitivity across the day. Our results underscore the crucial role of caregiver sensitivity in scaffolding infants' ability to regulate both over time and in the moment and highlight the value of accessing repeated measures of spontaneous caregiver behavior in everyday settings in contemporary work on infant physiological regulation.

## Conflicts of Interest

The authors declare no conflicts of interest.

## Data Availability

Data and code are not available on a public repository.
